# How Do Pelvic Parameters Correlate With Postoperative Outcomes When the Parameters Are Not Measured Preoperatively in Patients Undergoing Instrumented Lumbar Fusion?

**DOI:** 10.7759/cureus.16885

**Published:** 2021-08-04

**Authors:** Salah M Fallatah, Amjad M Altijani, Aisha A Alharbi, Safwan M Bourgleh, Mahdi M Bassi

**Affiliations:** 1 Orthopedic Surgery, Umm Al-Qura University, Makkah, SAU; 2 Orthopedic Surgery, The First Clinic, Jeddah, SAU; 3 Orthopedic Surgery, Dr. Soliman Fakeeh Hospital, Jeddah, SAU

**Keywords:** pelvic parameters, lumbar fusion, post-operative outcomes, qol

## Abstract

Objective: A normal age-adjusted sagittal alignment is an important factor in achieving long-term functional results after lumbar spinal fusion. We aim to determine if the changes in spino-pelvic alignment (SPA) correlate with post-operative functional outcomes in patients who underwent instrumented lumbar spine surgery when the parameters were not measured before.

Method: A retrospective review of medical records from 2012 to 2016, and radiographs of the patients who underwent instrumented fusion of the lumbar spine. The X-rays of the available preoperative lumbar spine were reviewed for SPA and compared with the last follow-up postoperative images. The patients were contacted by telephone to complete the EuroQoL 5 Dimensions 5-level questionnaire and visual analog scale for evaluation of their functional outcomes during 2017. Correlation studies were performed using Pearson’s coefficient.

Results: Forty-six patients were included with a mean age of 53 years and a follow-up of 47 months. There was a significant improvement in the functional outcomes and pain in the whole group. All the patients showed improvement in their SPA, and those who underwent more than two levels of fusion showed a significant improvement (P<0.05). Lumbar lordosis and sacral slope had a significant correlation with postoperative clinical improvement (R=0.8).

Conclusion: The study showed that single or double fusion has significant improvement in pain and functional outcome with a significant change in SPA.

## Introduction

Pelvic morphology has a direct influence on spino-pelvic balance of the human trunk in both physiological and pathological conditions [1]. Sagittal alignment is the most important radiological parameter to be restored in spinal deformity surgery and achieve positive functional outcomes after surgery to prevent long-term disability. Recent emphasis has been placed on the spino-pelvic parameters, namely pelvic incidence, sacral slope, pelvic tilt, and lumbar lordosis, to determine how they affect the outcomes of surgery [2]. Surgeons’ comprehensive understanding of these relationships is essential in enhancing postoperative outcomes [2]. Aoki et al. found a significant correlation between some of the radiological parameters and surgical outcomes [2]. These parameters are used as a reference for the evaluation of pelvic alignment [3,4]. An analysis of the pelvis in the sagittal plane is commonly assessed with three angular measurements, namely the pelvic tilt, the sacral slope, and the pelvic incidence [5].

Pelvic incidence (PI) is a key factor in sagittal balance analysis and is defined as the angle between the line perpendicular to the midpoint of the sacral plateau and the line from that point to the center of the femoral head. The average value of the incidence angle is 55° ± 10°. Pelvic tilt (PT) is the angle formed between the line drawn from the middle point of the sacral plateau to the center of the femoral head and the vertical line to the ground. When a person is standing, the mean value of PT is 13 ° ± 6 °. The sacral slope (SS) is the angle between the horizontal line and the sacral plateau. The pelvic incidence is the sum of the pelvic tilting angles with the sacral slope (PI = PT+SS) [5-7].

Lumbar lordosis (LL) varies by individual. LL is the upper plateau of the L1 vertebral body and the upper plateau of the sacrum (S1). LL can be measured using a simple formula (LL= PI ± 10 °) for age-adjusted calculation of a mismatch [2]. In order to optimally plan surgery for spinal instrumentation, the prediction of postoperative alignment is necessary [7]. Le Huec et al. showed that lumbar fusion, if not correctly performed, may result in loss of LL, increase in PT, decrease in SS, leading to spinal complications [8]. In addition, a PT increase was found to be correlated with persistent pain after spinal fusion [8]. Ren et al. [9] showed that there is a correlation between the effectiveness of surgery and changes in the spine-pelvic sagittal parameters before and after surgery. It has been suggested that single-level instrumented fusion is more forgiving compared to more than two levels of surgery when these parameters are not restored to the patient’s optimal alignment [9]. Patients with pelvic incidence-lumbar lordosis mismatch exhibit a 10-times higher risk of undergoing revision surgery than control patients if sagittal malalignment is present after lumbar fusion surgery [10]. The primary objective of this paper is to correlate the changes in these parameters with the postoperative functional outcome and pain relief (EQ-5D-5L and Visual Analogue Scale [VAS]).

## Materials and methods

**Figure 1:**
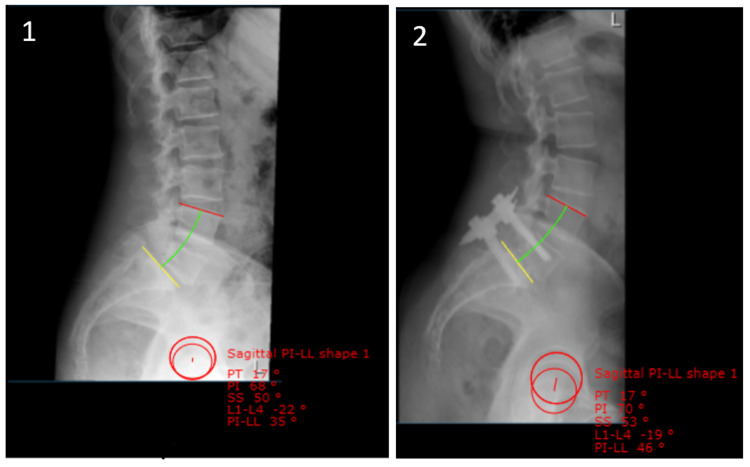
Pre-operative (1) and most recent post-operative (2) standing lateral pelvic radiographs.

This retrospective study was done in 2019 in Saudi Arabia. We retrospectively reviewed the medical records and radiological imaging of patients 18 years or older who underwent instrumented fusion of the lumbar spine (one or numerous levels between and including the first lumbar and sacral vertebrae) from 2014 to 2018. This study was conducted in two tertiary care centers for spine disorders after obtaining ethical approval from the Agency for Post-Graduate studies & Scientific Research of the faculty of medicine of Umm Al-Qura University, approval number HAPO-02-K-012-2018-01-250. The preoperative and the last follow-up postoperative standing AP and lateral pelvic radiographs were reviewed by two independent co-authors for measurement of spino-pelvic alignment (SPA) (pelvic tilt, sacral slope, pelvic incidence, and lumbar lordosis, L4/S1 angle) manually and using Surgimap software (Surgimap, Methuen, MA, USA) (Figure 1). The medical records were reviewed for patient demographic data, height, weight, and co-morbidities. The patients were contacted by phone during the year of the study to fill the EQ-5D-5L questionnaire to assess their quality of life and pain severity using the VAS. Patients younger than 18 years, with less than one year of follow-up, those with incomplete radiographs, or who could not be contacted were excluded from the study. Patients who refused to fill in the questionnaire were also excluded from the study.

After collecting all of the data, a statistical analysis was performed using SPSS software (IBM Corp., Armonk, NY, USA). Correlation studies were performed using Pearson’s coefficient to determine the relationship between radiological parameters and functional outcomes. Unpaired students’ t-tests were used to compare different instrumentation groups based on the number of levels fused. The level of significance level was set at 0.05.

## Results

Of 67 patients eligible to be included in the study, 46 had complete medical records and radiographs and agreed to fill in the EQ-5D-5L questionnaire. The mean follow-up time was 47 months (from 12-59 months). The mean age of the patients was 52.6 (from 29-80), 48% were male, and 52% were female. The mean body mass index (BMI) was 31.8 kg/m2 (SD 5.7) .59% of the patients in this study group were obese (BMI above 30). A summary of the mean values and range (both pre- and postoperatively) for each measurement of the SPA parameter is presented in Table 1. All parameters improved postoperatively. However, when the cohort just was divided into two groups, namely Group 1, which underwent one or two levels of fusion (20 patients), and Group 2, who underwent more than two levels of fusion (26 patients), postoperative improvement in parameters was only significant in Group 2 (P<.05). There was no difference between the measured and calculated PI (PI=PT+SS) in the cohort both pre- or post-operatively. The majority of patients in this study (90.5%) reported a significant improvement in pain after surgery (P <.05) on EQ5D5L with no significant correlation to SPA (57% were at level 5 preoperative and 90.5% at level 1-2 postoperatively). Overall, there was a statistically significant improvement in all dimensions (mobility, self-care, usual activities, pain/discomfort, and anxiety/depression), with the most improvement in pain/discomfort. The mean EQ-VAS score improved from 39 (21.3-59) before surgery to 83 (67- 99) postoperatively (P<.05). For the whole cohort, when SPA parameters were correlated with EQ-VAS score, lumbar lordosis and sacral slope had a significant correlation with EQ-VAS score (R=0.8).

**Table 1:** Spino-pelvic alignment (SPA) parameters, mean, range and standard deviation (SD) with normal values PT: Pelvic tilt, SS: Sacral slope, PI: Pelvic incidence, LL: Lumbar lordosis

		Mean	Range	SD	Normal values mean (range)
PT	Pre op	18.2	1-52	12	12 (3-18)
	Post op	20.3	1-56	13.1	
SS	Pre op	33.9	10-52	9.3	39 (32-49)
	Post op	30	5-47.3	9	
PI	Pre op	51.4	10-81	15.3	51 (43-62)
	Post op	49.3	19-90.4	15.7	
Calculated PI PI= PT+SS	Pre op	51.2	29-81	13.3	51(43-62)
	Post op	50.3	19-91	14.3	
LL	Pre op	37.9	10-65	11.9	+/- 10 of PI
	Post op	39.7	10-65	12.2	
L4-S1 Angle	Pre op	30.8	8.3-55.9	12	2/3 of LL value
	Post op	31.6	8.1-61.1	12.6	

## Discussion

Sagittal alignment restoration plays a role in offering positive functional outcomes after instrumentation in spinal surgery. SPA parameters are used to evaluate sagittal alignment, and significant abnormal changes in SPA during spinal fusion surgery may lead to poor outcomes [2]. This study found a direct correlation between pelvic position and patient self-reported function, whereas other studies have found that radiographic parameters are correlated to patient pain and disability [2,9,11,12]. To decrease these complications, the pelvic parameters should be measured pre-operatively with a pre-operative plan to have the abnormal variable parameters restored to near normal value during surgery [10]. The radiographic machines that allow 2D and 3D spinal assessment and accurate measurement of SPA are not widely available mainly due to cost. Furthermore, conventional radiographs are taken in a way that prevents taking these measurements. Therefore, this study is relevant and applicable in daily practice. The goal of the study was to investigate pre- and postoperative SPA parameters in a clinical setting when not measured preoperatively to compare with postoperative outcomes. The cohort, in general, showed a significant improvement in patients’ function and pain after surgery. The changes reported in SPA and its correlation with pain relief and outcomes are similar to those of other studies, which have that found restored the pelvic sagittal balance correlates with clinical outcomes [2]. This study’s findings are similar to those of Alqroom [13], who found that that single-level fusion has a minimal effect on pelvic parameters, whereas these changes are more significant for cases of more than two levels of fusion. However, Algroom found that pelvic tilt PT was correlated with outcomes. Different formulas have been proposed to achieve acceptable post-operative alignment related to outcomes and statistical models [13]. A sagittal vertical axis (SVA) of less than 50mm, pelvic tilt of less than 25°, and PI-LL less than 11° are associated with positive outcomes [11].

The published studies on functional outcome and SPA after spinal surgery have offered conflicting results. A number of studies have established a significant relationship between these parameters and outcomes, while others were unable to establish a relationship, even with positive outcomes, while these measurements were abnormal [2,3,7,10,12,14-16]. This inconsistency may be due to other factors contributing to the outcome not being studied or due to the short follow-up procedures. The sample of 46 patients is much higher than those of other studies on the same subject but it is still small. It has been suggested that there is no significant relationship between PI-LL and preoperative symptoms, yet a significant relationship has been found between PI-LL and the postoperative VAS score [2]. In addition, loss of lordosis was not always related to postoperative residual symptoms as other factors may play a role.

## Conclusions

This study is one of few that has looked at SPA and postoperative outcomes with the measurements not being taken preoperatively. The evaluation of lumbar lordosis and sacral slope, in addition to pelvic tilt and pelvic incidence, appear to be essential for longer fusion. Single or double fusion resulted in a significant clinical improvement with a significant change in SPA, whereas more than double fusion lead to significant changes in SPA. Furthermore, lumbar lordosis and sacral slope are most significantly correlated with the outcome. Similar to most spine research, the results of this study may change further investigation.
